# Supplementary material for: Contributions of mirror-image hair cell orientation to mouse otolith organ and zebrafish neuromast function
Source: bioRxiv. 2024 Sep 6:2024.03.26.586740. Preprint. [Version 2] doi: 10.1101/2024.03.26.586740 (PMC11398332; doi:10.1101/2024.03.26.586740)
Supplement: Supplement 1 [file NIHPP2024.03.26.586740v2-supplement-1.pdf]

bioRxiv preprint doi: https://doi.org/10.1101/2024.03.26.586740; this version posted September 6, 2024. The copyright holder for this preprint (which was not certified by peer review) is the author/funder, who has granted bioRxiv a license to display the preprint in perpetuity. It is made available under aCC-BY 4.0 International license.

Supplemental Figures

bioRxiv preprint doi: https://doi.org/10.1101/2024.03.26.586740; this version posted September 6, 2024. The copyright holder for this preprint (which was not certified by peer review) is the author/funder, who has granted bioRxiv a license to display the preprint in perpetuity. It is made available under aCC-BY 4.0 International license.

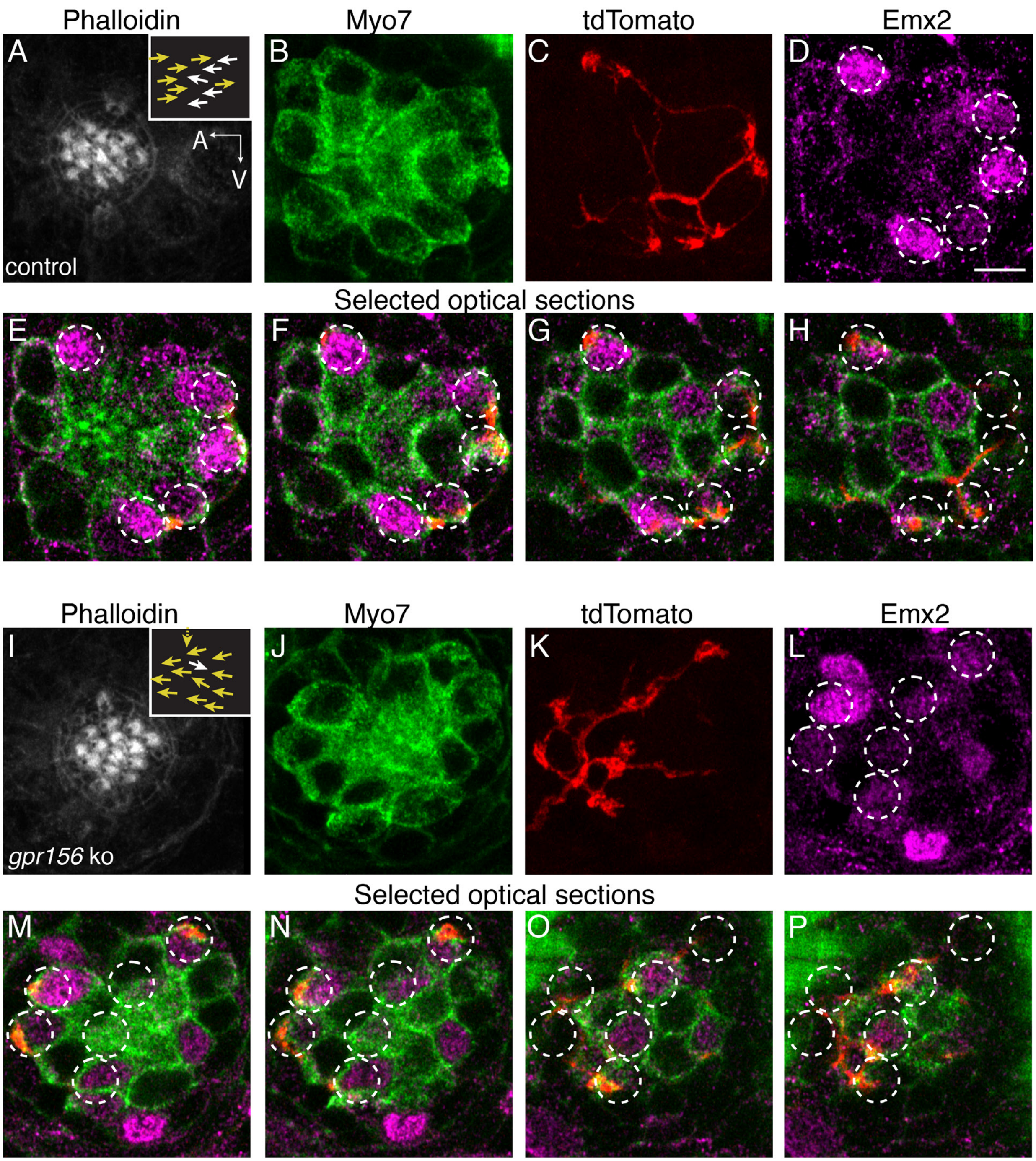

Supplemental figure 1. Gpr156 was not required for lateral-line afferents to select Emx2^{+} HCs. A-H) Images of a control neuromast innervated by a single afferent fiber that contacts Emx2^{+} cells. (A) Phalloidin label reveals 14 hair bundles (7 A>P and 7 P>A hair bundles). Z-stack projections show all HCs labeled with Myo7a (**B**) a single afferent fiber expressing tdTomato (**C**) and Emx2^{+} and Emx2^{-} HCs (**D**). (**E-H**) Selected optical sections

bioRxiv preprint doi: https://doi.org/10.1101/2024.03.26.586740; this version posted September 6, 2024. The copyright holder for this preprint (which was not certified by peer review) is the author/funder, who has granted bioRxiv a license to display the preprint in perpetuity. It is made available under aCC-BY 4.0 International license.

of (**B**–**D**) highlight the single afferent contacting individual $Emx2^+$ HCs. (**I**-**P**) Images of a *gpr156* mutant neuromast innervated by a single afferent fiber that contacts $Emx2^+$ cells. **I**) Phalloidin label revealed 14 hair bundles (12 A>P and 1 P>A hair bundles, 1 at 90°). Z-stack projections show all HCs labeled with Myo7a (**J**) a single afferent fiber expressing tdTomato (**K**) and $Emx2^+$ and $Emx2^-$ HCs (**L**). **M**-**O**) Selected optical sections of (**J**–**L**) highlight a single afferent in a *gpr156* mutants contacting individual $Emx2^+$ HCs. Arrows in **A** and **I** indicate the orientation of the hair bundles in each example. Scale bar: 5 μm.

bioRxiv preprint doi: https://doi.org/10.1101/2024.03.26.586740; this version posted September 6, 2024. The copyright holder for this preprint (which was not certified by peer review) is the author/funder, who has granted bioRxiv a license to display the preprint in perpetuity. It is made available under aCC-BY 4.0 International license.

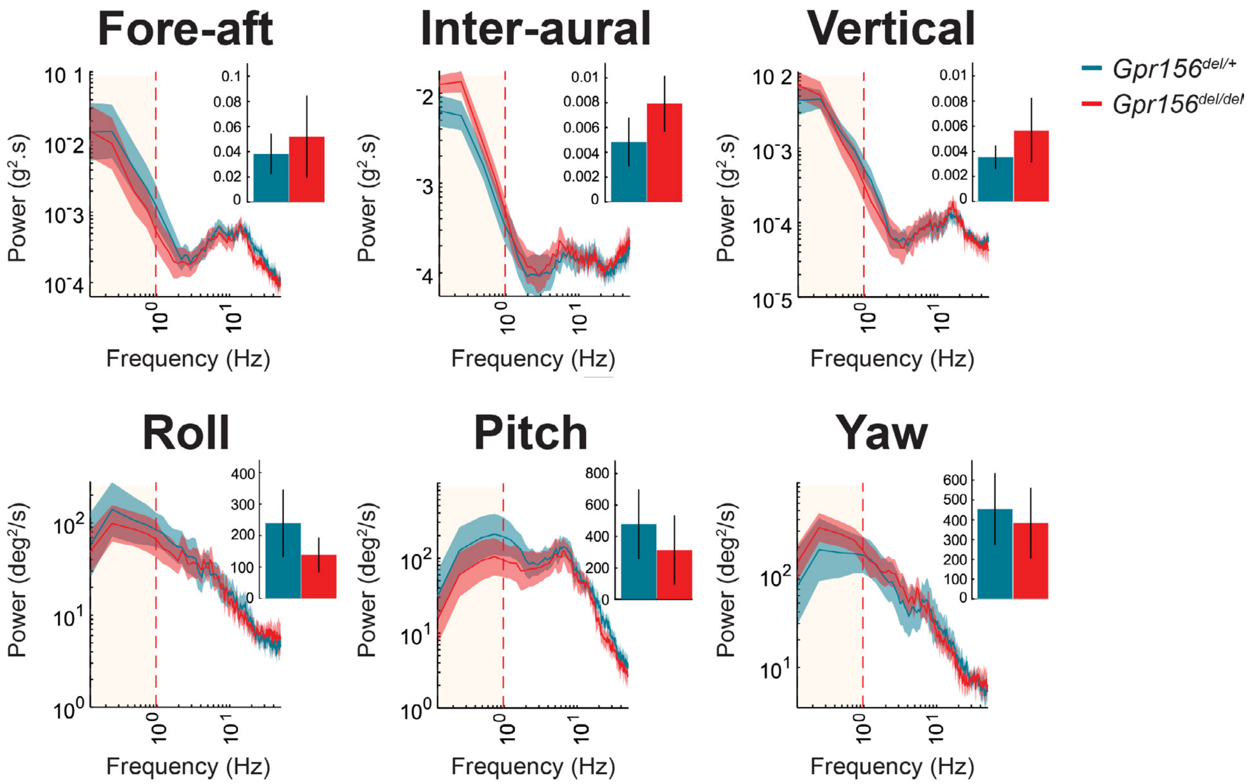

Supplemental figure 2. No resting head tremor was observed in *Gpr156$^{del/del}$* mice. Comparison of power spectra density of head movements at rest in translational axes and rotational axes between control and *Gpr156$^{del/del}$*. N is 8 and 7 for control and *Gpr156$^{del/del}$* mice respectively. Error bars: SEM.

bioRxiv preprint doi: https://doi.org/10.1101/2024.03.26.586740; this version posted September 6, 2024. The copyright holder for this preprint (which was not certified by peer review) is the author/funder, who has granted bioRxiv a license to display the preprint in perpetuity. It is made available under aCC-BY 4.0 International license.

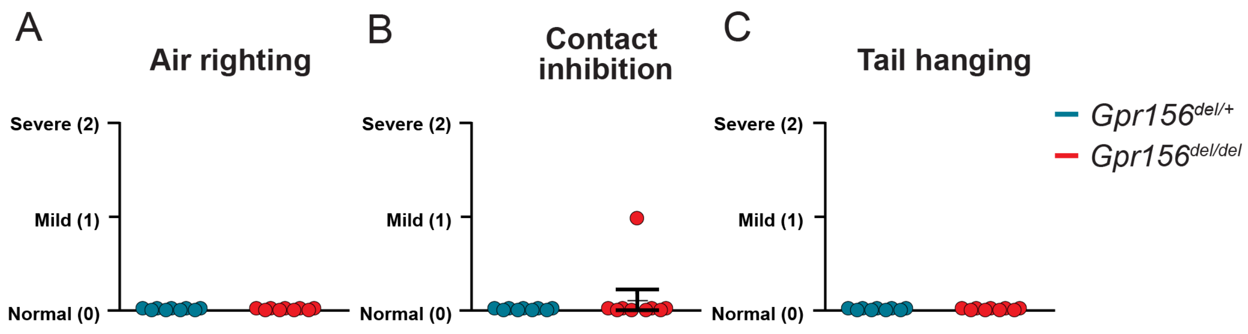

Supplemental figure 3. *Gpr156$^{del/del}$* mice scored normally on several basic standard behavioral tests. Comparison of assigned scores between *Gpr156$^{del/del}$* and control mice show no impairment in (**A**) air righting, (**B**) contact inhibition, (**C**) tail hanging. N is 9 and 7 for control and *Gpr156$^{del/del}$* mice respectively. Error bars: SEM.

bioRxiv preprint doi: https://doi.org/10.1101/2024.03.26.586740; this version posted September 6, 2024. The copyright holder for this preprint (which was not certified by peer review) is the author/funder, who has granted bioRxiv a license to display the preprint in perpetuity. It is made available under aCC-BY 4.0 International license.

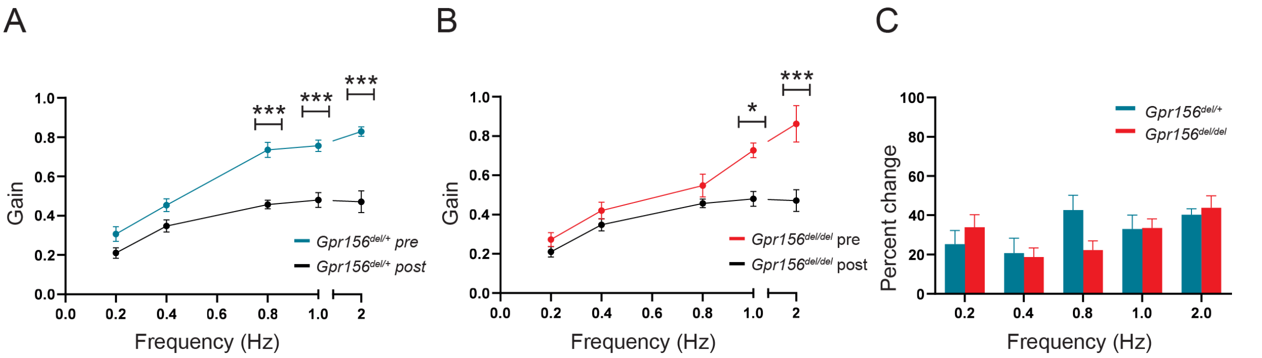

Supplemental figure 4. VOR learning was unimpaired in *Gpr156*$^{del/del}$ mice. A) VORd gain (mean ± SEM) before and after the VOR gain-down training plotted as a function of frequency for control mice (N = 7). **B**) VORd gain (mean ± SEM) before and after the VOR gain-down training plotted as a function of frequency for *Gpr156*$^{del/del}$ mice (N = 7). **C**) Percent change in VOR gain for control and *Gpr156*$^{del/del}$. *P<0.05, ***P<0.001.
